# Evolution of the fish heart by sub/neofunctionalization of an *elastin* gene

**DOI:** 10.1038/ncomms10397

**Published:** 2016-01-19

**Authors:** Yuuta Moriyama, Fumihiro Ito, Hiroyuki Takeda, Tohru Yano, Masataka Okabe, Shigehiro Kuraku, Fred W. Keeley, Kazuko Koshiba-Takeuchi

**Affiliations:** 1 Division of Cardiovascular Regeneration, Institute of Molecular and Cellular Biosciences, The University of Tokyo, 1-1-1 Yayoi, Bunkyo, Tokyo 113-0032, Japan; 2 Division of Ecological Genetics, National Institute of Genetics, 1111 Yata, Mishima, Shizuoka 411-8540, Japan; 3 Department of Genetics, The Graduate University for Advanced Studies (SOKENDAI), 1111 Yata, Mishima, Shizuoka 411-8540, Japan; 4 Department of Biological Sciences, Graduate School of Science, The University of Tokyo, 7-3-1 Hongo, Bunkyo, Tokyo 113-0033, Japan; 5 Department of Anatomy, The Jikei University School of Medicine, 3-25-8 Nishishinbashi, Minato, Tokyo 105-8461, Japan; 6 Phyloinformatics Unit, RIKEN Center for Life Science Technologies, 2-2-3 Minatojima-minamimachi, Chuo, Kobe, Hyogo 650-0047, Japan; 7 Research Institute, The Hospital for Sick Children, 555 University Avenue, Toronto, Ontario M5G 1X8, Canada; 8 Department of Biochemistry, University of Toronto, Toronto, Ontario M5S 1A8, Canada; 9 Department of Integrated Biosciences, Graduate School of Frontier Sciences, The University of Tokyo, 5-1-5 Kashiwanoha, Kashiwa, Chiba 277-8561, Japan

## Abstract

The evolution of phenotypic traits is a key process in diversification of life. However, the mechanisms underlying the emergence of such evolutionary novelties are largely unknown. Here we address the origin of bulbus arteriosus (BA), an organ of evolutionary novelty seen in the teleost heart outflow tract (OFT), which sophisticates their circulatory system. The BA is a unique organ that is composed of smooth muscle while the OFTs in other vertebrates are composed of cardiac muscle. Here we reveal that the teleost-specific extracellular matrix (ECM) gene, *elastin b*, was generated by the teleost-specific whole-genome duplication and neofunctionalized to contribute to acquisition of the BA by regulating cell fate determination of cardiac precursor cells into smooth muscle. Furthermore, we show that the mechanotransducer *yap* is involved in this cell fate determination. Our findings reveal a mechanism of generating evolutionary novelty through alteration of cell fate determination by the ECM.

## Abstract (web summary)

The bulbus arteriosus is an organ unique to the heart of teleosts, composed of specialized smooth muscle. Here, the authors show that the gene *elastin b*, which regulates cell fate of cardiac precursor cells into smooth muscle, evolved after whole-genome duplication and neofunctionalization in teleosts.

## Graphical abstract

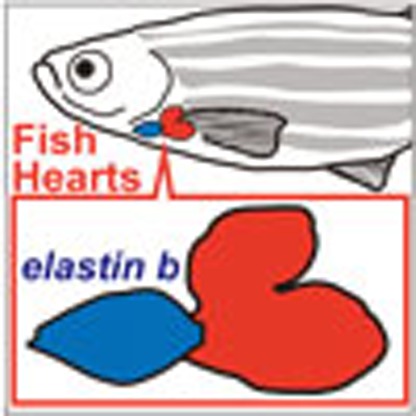

The *de novo* evolution of complex phenotypic traits is a key process in the diversification of life. However, investigation of genetic changes associated with these remains one of the greatest challenges in the field of evolutionary developmental biology^1^,^2^. Regressive losses of complex traits have received much attention^3^,^4^, but these kinds of studies offer limited insights into the constructive evolution of complex novel traits. Indeed, the sharpest critiques of Darwin's theory of evolution by natural selection often centred on explaining how novel body parts arose. In this context, investigations into the *de novo* evolution of complex phenotypic traits are open questions for evolutionary biology^5^,^6^,^7^,^8^. Recent studies have unravelled the genetic causes for generating evolutionary novelties and suggested that new developmental programs emerge mostly through co-option of pre-existing regulatory gene networks via changes in their regulation and deployment (‘old genes playing new tricks')^5^. However, it still remains largely unknown whether ‘genetic novelties (new genes)' directly contribute to ‘phenotypic novelties', and this is one of the primary goals of evolutionary developmental biology (evo–devo) research.

Whole-genome duplication (WGD) is a phenomenon which has contributed to emergence of evolutionary novelties by supplying genetic materials^9^,^10^,^11^. In his milestone book, *Evolution by Gene Duplication*^11^, Susumu Ohno put forward the hypothesis that genome duplication and subsequent subfunctionalization and neofunctionalization are a potent force generating phenotypic evolution. However, our current knowledge is limited to the fact that neofunctionalization is a very rare event in comparison with subfunctionalization, and the most likely fate of duplicated genes is pseudogenization, in which one of the duplicated copies is silenced. Thus the relationship between WGDs and phenotypic evolution is still elusive^12^,^13^,^14^,^15^.

The bulbus arteriosus (BA) is a specialized organ in the outflow tract (OFT) of teleost heart, and is regarded as an evolutionary novelty in the teleost lineage^16^. The BA has an important role of acting as a ‘windkessel' organ, absorbing the energy of a bolus of blood ejected by the ventricle by elastic expansion and recoil and smoothing the pressure wave down the arterial tree^17^,^18^. Anatomical and histological characteristics of the BA are quite different from those of other vertebrate OFTs. The BA is composed of smooth muscle and is rich in elastin, while other vertebrates OFTs are composed of cardiac muscle lined by a thin elastin layer. The abundance of elastic fibres in the BA is considered to have a central role for its effective physiological function^17^,^18^,^19^.

Although the morphology of OFTs has been reported in several actinopterygian species, the phylogenetic timing of emergence of the BA is still elusive^16^,^17^,^18^,^20^,^21^,^22^,^23^,^24^,^25^,^26^,^27^,^28^,^29^,^30^. Furthermore, genetic and developmental mechanisms underlying generation of the BA are largely unknown; how did/do teleost species, in their evolution and development, convert cell types of the BA from cardiac to smooth muscle cells and accumulate specialized elastic fibres? Interestingly two *elastin* genes, designated *elastin a* (*elna*) and *elastin b* (*elnb*), were initially identified in the zebrafish (*Danio rerio*) genome, compared with a single gene in other vertebrate genomes^31^. Furthermore, it was reported in both yellowfin tuna^17^,^18^ and rainbow trout^32^ that the architecture and properties of elastin purified from BA was distinct from that of the ventral aorta, with a decreased elastic modulus and increased viscoelasticity. These characteristics are consistent with an increase in the average size of the hydrophobic domains of elastin b (Elnb) compared with elastin a (Elna)^33^. These facts raise the possibility that the windkessel function of BA in teleosts is linked to the presence of Elnb.

Here we address the genetic and developmental basis underlying BA formation and deduce the evolutionary path of acquisition of the BA in vertebrate evolution. We reveal that the BA is indeed an evolutionary novelty in the teleost lineage, and that *elnb*, which was generated by duplication of the *elastin* gene in the teleost-specific 3rd round WGD (3R WGD), is involved in morphogenesis of BA. We provide evidence that *elnb* was subsequently neofunctionalized, acquiring a function to regulate cell fate determination of BA cells into smooth muscle cells. Furthermore, we found that the mechanotransducer gene *yap* is involved in the process of cell fate determination in BA. These facts suggest that alteration of the extracellular environment by a novel extracellular matrix (ECM) generated evolutionary novelty by changing cell fate determination through mechanotransduction. Taken together, our results clearly illustrate that the 3R WGD and subsequent sub/neofunctionalization of *elastin* genes contributed to generation of the evolutionary novel BA, and to the explosive radiation under natural selection in the teleost lineage.

## Results

### The phylogenetic timings of BA acquisition and the 3R WGD

**Figure 1:**
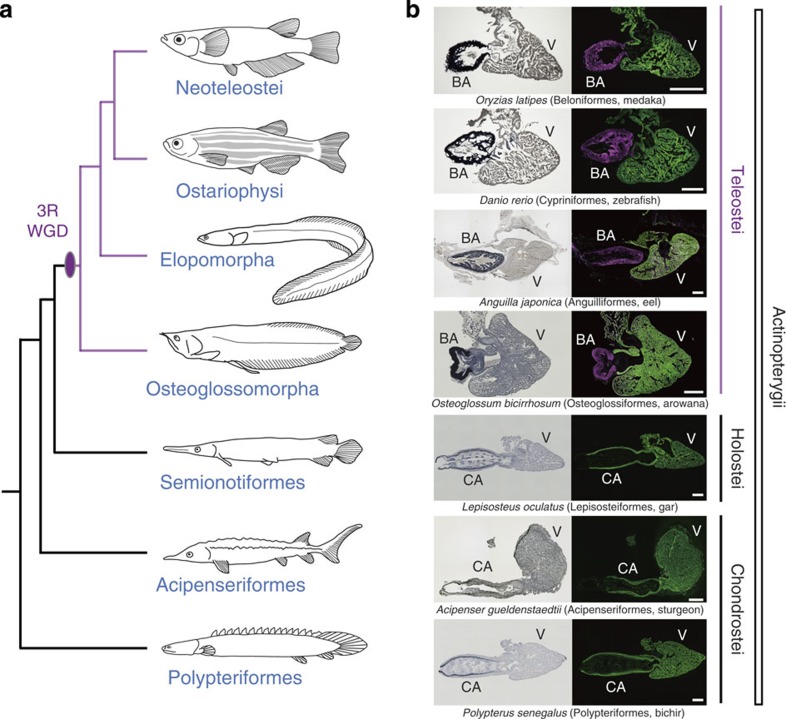
Anatomy and histology of OFTs in actinopterygians. (**a**) Phylogeny of actinopterygian species. (**b**) Anatomy and histology of OFTs in representative actinopterygian species. Left panels are results of Elastica van Gieson staining, which visualizes the accumulation of elastic fibres. Note that the elastic fibres of BAs in teleosts are abundant, while those of CAs in non-teleost fishes are restricted to the inner lining. Right panels are results of double immunohistochemistry against α-sarcomeric actinin (cardiac muscle, green) and myosin light-chain kinase (smooth muscle, magenta). Note that teleost BAs are composed of smooth muscle, while CAs in non-teleost fish are composed of cardiac muscle. The phylogenetic timing of acquisition of BA is coincident with 3R WGD. CA, conus arteriosus; V, ventricle. Scale bars, 400 μm.

It has been reported that the OFT in some non-teleost fish consists of myocardium, and exhibits variable numbers of valves inside the lumen^34^,^35^,^36^. This type of OFT is called a ‘conus arteriosus' and is regarded as an ancestral characteristic of the vertebrate OFT^34^,^35^,^36^,^37^,^38^. To determine whether the BA is indeed an evolutionary novelty exclusive to the teleost lineage, we confirmed the phylogenetic timings of emergence of the BA in representative actinopterygian fish by anatomical and histological analyses using Elastica van Gieson staining and cardiac- and smooth muscle-specific antibodies (α-sarcomeric actin, green; myosin light-chain kinase, magenta). We found that the OFTs of Chondrostei (Polypteriformes; *Polypterus senegalus* and Acipenseriformes; *Acipenser gueldenstaedtii*) and Holostei (Semionotiformes; *Lepisosteus oculatus*) were composed of myocardium with a thin elastin layer inside the lumen. In contrast, OFTs of Teleostei (Osteoglossomorpha, *Osteoglossum bicirrhosum*; Elopomorpha, *Anguilla japonica*; Ostariophysi, *Danio rerio*; Neoteleostei, *Oryzias latipes*) were elastin-rich and composed of smooth muscle, which are the features of the BA (Fig. 1a,b). Anatomical and histological analyses showed that the OFTs of actinopterygian fish were markedly different before and after the 3R WGD (Fig. 1). These results are consistent with previous reports^20^,^21^,^22^,^23^,^24^,^25^,^26^,^27^,^28^,^29^,^30^, and clearly show that the phylogenetic timing of BA acquisition is linked to the 3R WGD. These data suggest that BA would have been acquired as a result of 3R WGD during teleost evolution.

### Subfunctionalization of an *elastin* gene in teleosts

**Figure 2:**
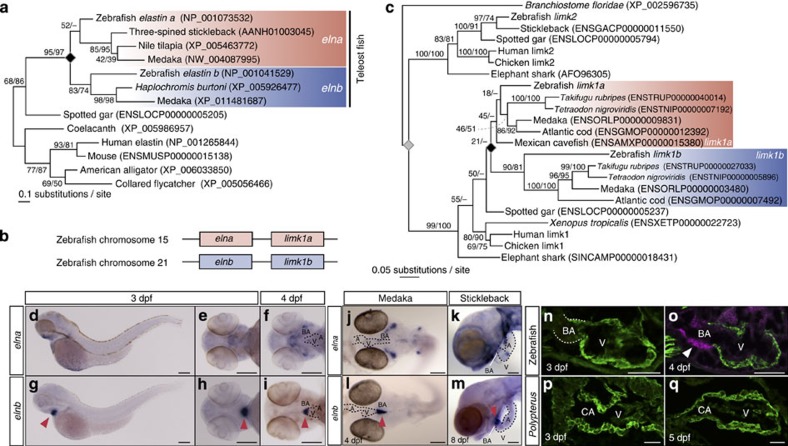
Molecular phylogeny and expression patterns of *elna* and *elnb* in teleost. (**a**) Molecular phylogenetic tree of *elastin* genes (*elna/elnb*) inferred using 83 amino-acid sites (shape parameter of the gamma distribution alpha=0.67). The gene duplication between *elna* and *elnb* is indicated with the black diamond. (**b**) Syntenic relationship of *elna/elnb* and *limk1a/1b*. (**c**) Molecular phylogenetic tree of *limk1* genes inferred using 303 amino-acid sites (alpha=1.03). The gene duplication between *limk1a* and *limk1b* is indicated with the black diamond, while a more ancient gene duplication is shown with the grey diamond. The *limk1a-1b* duplication is shown to have occurred early in the teleost fish lineage, coinciding with 3R WGD. (**d**–**i**) Expression patterns of *elna* and *elnb* in zebrafish embryos at 3 dpf (**d**,**e**,**g**,**h**) and 4 dpf (**f**,**i**). Arrowheads indicate signals in the BA. Scale bars, 200 μm. (**j**–**m**) Expression patterns of *elna* and *elnb* in medaka (**j**,**l**) and stickleback (**k**,**m**) embryos. Note that *elnb* is expressed only in the BA in both medaka and stickleback embryos. Scale bars, 200 μm. (**n**–**q**) Double immunohistological staining of developing OFTs in zebrafish (**n**,**o**) and *Polypterus* (**p**,**q**) embryos against α-sarcomeric actinin (cardiac muscle, green) and myosin light-chain kinase (smooth muscle, magenta). Smooth muscle cells appear in the zebrafish BA at 4 dpf, but could not be detected in the *Polypterus* CA. A, atrium. Scale bars, 50 μm.

Next we confirmed whether two *elastin* paralogues were generated by the 3R WGD by molecular phylogenetic analysis. The resultant phylogenetic tree showed that *elna* and *elnb* were duplicated after the split of the spotted gar (Semionotiformes, a non-teleost fish, Fig. 2a, black diamond). This phylogenetic timing is linked to that of 3R WGD. However, *elastin* phylogeny is limited in its taxonomic coverage and alignment sequence length, so next we scanned flanking regions in the genome for further confirmation. This led to the identification of *LIM domain kinase* (*limk*) *1a* and *1b* genes as neighbours of *elna* and *elnb*, respectively, which should allow higher resolution with more unambiguously aligned sites (Fig. 2b). The phylogenetic tree of *limk1a* and *limk1b* in various vertebrates revealed that the duplication of these genes also occurred after the split of the spotted gar (Fig. 2c, black diamond). Taken together, these results indicate that genomic locus including *eln* and *limk1* genes was duplicated in the 3R WGD.

To understand the molecular mechanism underlying BA formation, we examined the expression patterns of *elna* and *elnb* in developing teleost embryos. In zebrafish, we found that *elnb* expression was initiated at 3 days post-fertilization (dpf) in the BA in a region-specific manner, and the expression was intensified at 4 dpf (Fig. 2g–i, arrowheads). In contrast, *elna* was expressed not only in the BA but also other tissues, such as the cranial skeleton and swim bladder (Fig. 2d–f and Supplementary Fig. 2a). These results are consistent with previous reports^31^. Next we examined the expression patterns of *elna* and *elnb* in other teleost species to investigate whether expression patterns of *elna* and *elnb* are conserved, using medaka (*Oryzias latipes*) and threespine stickleback (*Gasterosteus aculeatus*) embryos. We found that *elnb* expression patterns were restricted to the BA, while *elna* was observed in various tissues in both medaka (Fig. 2j,l) and stickleback (Fig. 2k,m), similar to zebrafish. The differential expression patterns of *elna* and *elnb* in teleosts suggest that subfunctionalization occurred in these genes, and the restricted expression of *elnb* in the BA suggests that *elnb* may be involved in organogenesis of the BA.

To clarify the relationship between the kinetics of *elnb* expression and BA morphogenesis in heart development, we examined the timing of smooth muscle cell differentiation in the OFT region. We found that smooth muscle cells appeared at 4 dpf, just after the onset of *elnb* expression (Fig. 2n,o, arrowhead). Furthermore, we studied OFT development in *Polypterus senegalus* embryos (Polypteriformes, a non-teleost fish), whose OFT is composed of cardiomyocytes even in the adult (Fig. 1). The *Polypterus* embryonic OFT was also composed of cardiomyocytes at 3 and 5 dpf, and smooth muscle cells were never observed in the OFT region (Fig. 2p,q). To examine the expression pattern of the *eln* gene in non-teleost fish possessing cardiac OFT, we isolated the *Polypterus elastin* gene and performed *in situ* hybridization in *Polypterus* embryos. As expected, *Polypterus* had one *elastin* gene, and its expression was observed in various tissues including OFT, which is similar to that of zebrafish *elna* (Supplementary Fig. 1). These results suggest that *elnb* expression is tightly related to smooth muscle differentiation in the OFT both in development and evolution.

### *elnb* was neofunctionalized and involved in BA morphogenesis

**Figure 3:**
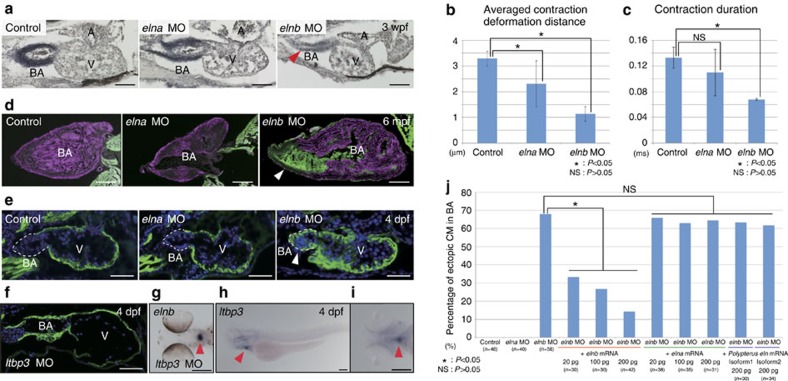
Functions of *elna* and *elnb* in BA development. (**a**) Morphology of BA and elastin accumulation in *elna* and *elnb* zebrafish morphants at 3 weeks post-fertilization (wpf). Elastica van Gieson staining highlights hypoplasia and decreased elastin accumulation in *elnb* morphant BA (arrowhead). Scale bars, 50 μm. (**b**) Averaged contraction deformation distances in control, *elna* and *elnb* morphant BAs. Averaged contraction deformation distances in the *elna* and *elnb* BA are significantly reduced compared with that of the control. *n*=4 each. *P<0.05, NS: *P*>0.05 (**c**) Contraction durations in control, *elna* and *elnb* morphant BAs. Contraction duration in *elnb* BA is greatly reduced compared with that of the control. *n*=4 each. *P<0.05, NS: *P*>0.05 (**d**) Anatomy and histology of BA in control, *elna* and *elnb* morphants at 6 months post-fertilization (mpf) using double immunohistochemistry; α-sarcomeric actinin (cardiac muscle, green) and myosin light-chain kinase (smooth muscle, magenta). Ectopic cardiomyocytes are observed in *elnb* morphant BA (arrowhead). Scale bars, 200 μm. This is also the case with embryonic hearts (**e**). Scale bars, 50 μm. (**f**) Anatomy and histology of BA in *ltbp3* morphants at 4 dpf. Ectopic cardiomyocytes are observed in *ltbp3* morphant BA with deformation of heart morphology. Scale bar, 50 μm. (**g**) *elnb* expression pattern in *ltbp3* morphants at 4 dpf. Expression pattern of *elnb* is not altered in *ltbp3* morphants. Arrowhead indicates *elnb* expression. Scale bar, 200 μm. (**h**,**i**) *ltbp3* expression pattern at 4 dpf embryos. *ltbp3* is expressed in the BA (arrowheads). Scale bars, 200 μm. (**j**) Functional divergence of *elna* and *elnb* in morphogenesis of the BA. Rescue experiment of *elnb* morphants by injection of *elna, elnb* and *Polypterus eln* full-length mRNAs. Percentages of ectopic cardiomyocytes in the BA are shown in each condition. *elna* and *Polypterus eln* mRNA did not rescue the *elnb* morphant phenotypes while *elnb* mRNA did. *P<0.05, NS: *P*>0.05. Dashed lines depict outlines of the BA. Values are reported as mean±s.d. and *P* values were determined by the *t*-test. NS, not significant.

To elucidate the exact function of *eln* genes for smooth muscle differentiation and BA formation, we performed knockdown experiments using antisense morpholino oligonucleotides (MOs) in zebrafish embryos. Three weeks after injection of MOs (4 ng each), sections were prepared to examine Eln synthesis and BA morphology. The *elnb* morphants exhibited severe hypoplasia of the BA and decreased elastin accumulation, while *elna* morphants exhibited no obvious defect in BA morphology (Fig. 3a). *elna* morphants exhibited a swim bladder defect, where *elna* expression level is relatively high (Supplementary Fig. 2a,b). To characterize the effect of *elna* and *elnb* knockdown on BA formation and function, we quantified the motions of each morphant BA cells by using the motion vector prediction method, which quantifies cell movement in a non-invasive manner^39^,^40^. For motion vector prediction analysis, we used mild phenotype morphants (2 ng each) to reduce secondary effects. This small dose of *elnb* MO resulted in normal morphology of the BA. As a result, the averaged contraction deformation distances were significantly reduced in *elna* and *elnb* morphant BA cells (Fig. 3b). The contraction duration of *elna* morphants was slightly reduced but not significant, while that of *elnb* morphants was greatly reduced compared with those of control embryos (Fig. 3c). These results suggest that *elna* and *elnb* are involved in the function of BA, and Elnb is likely a major contributor to BA elasticity, which contributes significantly to its function^16^,^17^,^18^.

The *elna* and *elnb* morphants could survive and grow to adults under mild conditions (2 ng MO injection). We performed anatomical and histological analyses to examine the developmental effects of *elna* and *elnb* knockdown. Surprisingly, we found that ectopic cardiomyocytes were induced in the BA treated with *elnb* MO at 6 months after injection (Fig. 3d, arrowhead). Further we confirmed that ectopic cardiomyocytes were observed in the BA in the embryonic hearts at 4 days after injection (Fig. 3e, arrowhead). Such ectopic cardiomyocyte formation in the BA was observed only in *elnb* morphants and not in *elna* morphants. Next, we confirmed the phenotypes of genetic mutants of *elna* and *elnb*, which were generated by CRISPR/Cas9 system and possessed the mutations to each coding sequences. We observed ectopic cardiomyocytes in the BA of *elnb* genetic mutant embryos similar to those of morpholino knockdown embryos (Supplementary Fig. 3a,b). It was reported that latent TGF-β binding protein 3 (*ltbp3*) marks the second heart field (SHF) in zebrafish heart development, and embryos with *ltbp3* knocked down did not exhibit Elnb expression in the OFT region^41^. We performed histological and anatomical analyses in *ltbp3* knockdown embryos and found that cardiomyocytes were ectopically formed in the hypomorphic BA (Fig. 3f). However, *elnb* gene expression in the BA was not altered in *ltbp3* morphants (Fig. 3g). We confirmed the exact expression kinetics of *ltbp3* in heart development and found that *ltbp3* was expressed in the BA at 3 and 4 dpf (Fig. 3h,i and Supplementary Fig. 4). It has been reported that some *ltbps* regulate elastic fibre assembly of elastin by interacting with fibulin in mammalian skin and lung cells^42^,^43^,^44^. These results indicate that *ltbp3* does not affect proto-*elnb* production but is required for the maturation and function of Elnb.

To determine whether neofunctionalization occurred between *elna* and *elnb* genes, we designed a rescue experiment. We injected *elastin* MO and mRNA under the following conditions: (1) *elnb* MO and *elnb* full-length mRNA; (2) *elnb* MO and *elna* full-length mRNA; (3) *elnb* MO and *Polypterus eln* full-length mRNA in zebrafish embryos. When we injected *elnb* MO, 67.9% of embryos formed ectopic cardiomyocytes in the BA. In contrast, with a combination of *elnb* MO and *elnb* full-length mRNA, the rate of ectopic cardiomyocyte formation was decreased in a dose-dependent manner. This suggests that *elnb* mRNA rescues the *elnb* morphant phenotype. In contrast to this, injection of *elnb* MO and *elna* full-length mRNA did not rescue the *elnb* morphant phenotype (Fig. 3j). To examine whether the function of *elnb* is novel or not, next we cloned the *Polypterus eln* gene and conducted rescue experiment by injection of *elnb* MO and *Polypterus eln* mRNA. In the situation of subfunctionalization (the functions of *elna* and *elnb* in teleosts share that of ancestral *eln*) *Polypterus eln* mRNA would be able to rescue the *elnb* morphant phenotype. In contrast to this, in the situation of neofunctionalization (the function of *elnb* in teleost is different from that of ancestral *eln*; *elnb* acquired the new function), *Polypterus eln* mRNA would not be able to rescue the *elnb* morphant phenotype. We cloned two isoforms of *Polypterus eln* transcripts (isoform1 and isoform2) and we found that both of these *Polypterus eln* mRNAs did not rescue the *elnb* morphant phenotype (Fig. 3j). These results indicate that the function of *elnb* is distinct from those of *elna* and ancestral *eln*; neofunctionalization likely occurred between these two paralogues.

### *elnb* regulates cardiac/smooth muscle differentiation

**Figure 4:**
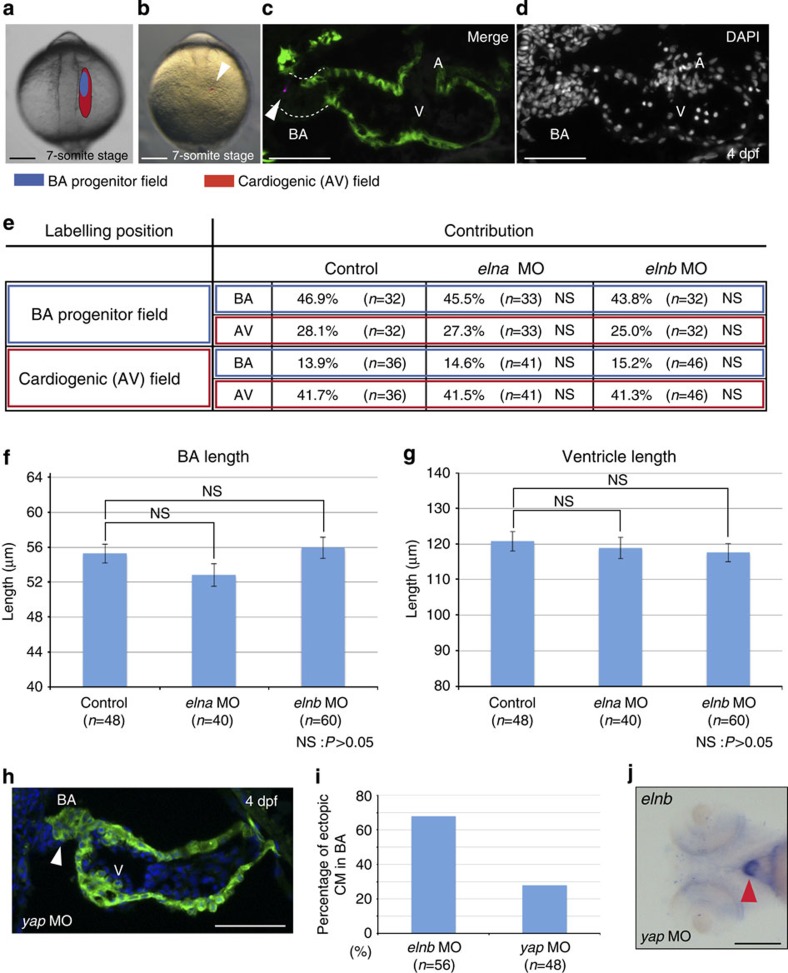
Cell fate determination is altered in *elnb* morphant BA. (**a**,**b**) Fate map of BA (blue) and cardiogenic (red) progenitors in anterior lateral plate mesoderm according to Hami *et al.*^45^ Scale bars, 200 μm. (**c**,**d**) Example of fate mapping result by injection of fluorescent tracer DiI. Arrowhead indicates injected DiI. Scale bars, 50 μm. (**e**) Result of cell lineage tracing experiment. In all cases, BA progenitor field to BA or cardiogenic and cardiogenic progenitor field to BA or cardiogenic in both *elna* and *elnb* morphants, no statistically significant differences were observd. NS: *P*>0.05. (**f**,**g**) Lengths of BA and ventricle in control, *elna* and *elnb* morphants. In all cases no statistically significant differences were observed. NS: *P*>0.05. (**h**) Anatomy and histology in *yap* morphant hearts at 4 dpf. Ecotopic cardiomyocytes are observed in BA (arrowhead). Scale bar, 50 μm. (**i**) Percentage of ectopic cardiomyocytes in the BA of *elnb* morphants (control) and *yap* morphants. (**j**) *elnb* expression in *yap* morphants. *elnb* expression is not changed in *yap* morphants (arrowhead). Scale bar, 200 μm. Values are reported as mean±s.d. and *P* values were determined by the *t*-test.

As described above, we observed ectopic cardiomyocytes in the BA of *elnb* morphants (Fig. 3d,e). To explain this phenotype, we designed two hypotheses; *elnb* regulates (1) migration pattern of cardiac precursor cell, or (2) fate determination of BA cells. To test this, we examined cell migration patterns of both BA and ventricle precursor cells. First, we conducted cell lineage tracing experiments by injection of the fluorescent tracer DiI. We injected DiI into the BA or cardiogenic (atrium or ventricle) progenitor fields at the 7-somite stage, as previously reported (Fig. 4a,b)^45^, and traced the contributions of DiI at 4 dpf (Fig. 4c,d). Contribution ratios of the BA field to the BA, the BA field to cardiogenic, the cardiogenic field to the BA and cardiogenic field to cardiogenic were not significantly changed in either *elna* or *elnb* morphants compared with the control (Fig. 4e, *P*>0.05). We next measured the lengths of the BA and ventricle because it was previously reported that the sizes of the BA and ventricle change reciprocally when the proportionate contribution of these common precursor cells from SHF is altered^45^. We found that differences in the lengths of the BA and ventricles were not significant between control and *elna* and *elnb* morphants (Fig. 4f,g, *P*>0.05). It was reported that phosphorylation levels of Smad1/5/8 in the arterial pole at 30 hours post-fertilization (hpf) is important for regulation of the proportionate contribution of common precursors from the SHF to the BA and ventricle^45^. We assumed that the phosphorylation level of Smad1/5/8 would be downregulated in *elnb* morphants if the migration of common precursors was affected. We examined the phosphorylation levels of Smad1/5/8 in *elna* and *elnb* morphants and found that the levels of pSmad1/5/8 signal were not changed from those of the control at 30 hpf (Supplementary Fig. 5a). Furthermore, we confirmed that *elnb* was not expressed in the BA at 30 hpf, suggesting that *elnb* is independent of phosphorylation levels of Smads (Supplementary Fig. 5b). Taken together, these results show that *elnb* regulates the cell fate determination of cardiac precursor cells in the BA, not their migration patterns. Elnb promotes differentiation of precursor cells into smooth muscle cells, and in the case where *elnb* expression is decreased or lost, the cardiac precursor cells differentiate into cardiomyocytes in the BA. The next question was how *elnb* regulates cell fate determination. From our experiments, we hypothesized that mechanotransduction might underlie BA cell fate determination because Elnb is a component of ECM that provides mechanical stress to cells. We focused on YAP, a mechanotransducer that senses mechanical signals and changes cell fate determination^46^. To test this, we knocked down and genetically mutated *yap* and found ectopic cardiomyocytes in the BA of *yap* morphants and mutants similar to *elnb* morphants (Fig. 4h,i and Supplementary Fig. 3c). Furthermore, we found that *elnb* expression in the BA was not altered in *yap* morphants (Fig. 4j). These results suggest the possibility that *yap* is involved in cell fate determination of BA cells through mechanotransduction.

## Discussion

In this study, we revealed the genetic and developmental basis underlying morphogenesis of the BA, an evolutionary novelty in the teleost lineage that sophisticates their single circulatory system and contributes to their explosive radiation in aquatic environment^16^,^34^,^37^,^38^,^47^.

We first confirmed the phylogenetic timings of emergence of the BA in actinopterygian evolution and found that only teleost species have BA (Fig. 1). This result indicates that the BA is a synapomorphy of teleost lineage. The OFT in fish heart, which is located between the ventricle and the ventral aorta, has received different names in the history of zoology and this persists today. It has variously been called ‘conus arteriosus', ‘bulbus arteriosus', ‘bulbus aortae', ‘truncus arteriosus' and even ‘bulbus cordis'^16^,^35^,^36^,^48^. It has been generally assumed that in most teleosts the conus arteriosus is vestigial or even absent coincident with the remarkable development of the BA in this zoological group^47^,^49^,^50^,^51^. Recent studies reported the existence of BA in the non-myocardial portion of the conus arteriosus in elasmobranch species hearts^22^,^52^, where is covered by epicardium and is crossed by the coronary arterial trunks. However, the structures in these non-myocardial portions are quite different from those of BA seen in the teleost, and similar to those of non-teleost fishes (Fig. 1). Thus, it is not plausible to regard these as ‘BA' or a ‘homologous organ to BA'. These caused further confusion in this field. Given this situation, here we would like to redefine the ‘BA' as a teleost-specific organ with a large amount of elastic fibres and composed of smooth muscle cells, which may be endowed by Elnb. Our findings strongly indicate that BA is a synapomorphy in the teleost lineage (Figs 1 and 2).

We revealed that neofunctionalization had occurred among *eln* genes in teleost evolution (Fig. 3). Gene duplication and subsequent subfunctionalization and neofunctionalization have been often addressed by focusing on the phenomena of teleost-specific 3R WGD^53^. However, the consequences of the 3R WGD, especially its impact on the massive radiation of teleosts in the aquatic environment, have been matter of controversial debate. Although the WGDs are regarded as a potent force providing novel genes for evolutionary adaptation and innovation, it is less clear whether the 3R WGD was directly linked to the acquisition of evolutionary novelty in infraclass Teleostei. Our results demonstrated that the 3R WGD and subsequent neofunctionalization of *eln* genes contributed to acquisition of BA, which is believed to be one of the most important evolutionary novelties for adaptive radiation in the aquatic environment. Our study sheds light again on the importance of gene duplication for evolution, as proposed by Susumu Ohno in his book *Evolution by Gene Duplication*^11^. In addition to the previous study that revealed convergent evolution of electric organ in Mormyridae and Gymnotiformes driven by neofunctionalization of *Scn4a* gene^54^, our study clearly illustrates that WGD and subsequent neofunctionalization contributes to generation of phenotypic evolutionary novelty under natural selection and the teleost-specific 3R WGD demarcates infraclass Teleostei.

Our results are highly suggestive that elastogenesis of Elnb is regulated by *ltbp3*, which is known to be a SHF marker in zebrafish^41^. It is reported that some *ltbps* regulate elastic fibre assembly of elastin^42^,^43^,^44^, and these facts raise the possibility that assembly of Elnb in the BA would be regulated by *ltbp3*. However, not only teleosts but also other vertebrates have *ltbp3* in their genomes^55^. In this context, it is likely that *ltbp3* is expressed in the OFT in basal actinopterygians, followed by *elnb* expression in the OFT region after the 3R WGD, and then elastogenesis would be promoted by *ltbp3*. The expression patterns of *ltbp3* in non-teleost fish await further study, and these results will contribute to further understanding of the origin of the SHF in vertebrate evolution.

Our results are also suggestive of an important role of Elnb in the fate determination of BA cells. From our cell lineage tracing experiments, we provide strong evidence that Elnb regulates cell fate determination of BA cells into smooth muscle cells (Fig. 4). Organogenesis and morphogenetic movements involve dynamic remodelling of ECM scaffolds in the embryo. In addition to physical connections of cells within tissues, ECMs act as the three-dimensional elastic scaffolds that resist cell-traction forces and thereby regulate tissue development by altering physical force distributions, changing the cellular force balance and modulating cell shape^56^. Previous studies have indicated that material properties of polymeric elastin from BA and ventral aorta differ in the teleost, with BA elastin showing a decreased elastic modulus and increased viscoelasticity^17^,^18^,^19^. The different functions between Elna and Elnb, reported here, are consistent with these facts (Fig. 3b,c). Further we found that the mechanotransducer *yap* was involved in the process of cell fate determination of BA cells (Fig. 4h). These facts suggest that the different elasticity and stiffness of Elnb in BA affects organogenesis of BA by regulating the cell fate determination coordinating with mechanotransduction. This is the first report that elastin, a type of ECM, regulates the cell fate of cardiac progenitor cells.

**Figure 5:**
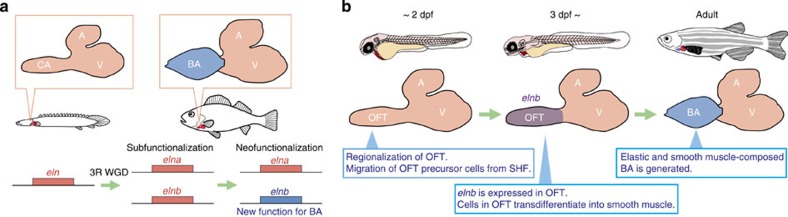
3R WGD and subsequent sub/neofunctionalization of an *elastin* gene contributed to acquisition of BA in teleost evolution. (**a**) Subfunctionalization and neofunctionalization of *elastin* genes in actinopterygian evolution. The 3R WGD duplicated *elastin* into *elna* and *elnb* and subfunctionalization occurred between these two genes. After duplication of these genes, mutations were accumulated in the gene body of *elnb*, and *elnb* acquired a new function for BA morphogenesis; neofunctionalization occurred. (**b**) Morphogenesis of BA by *elnb* in zebrafish heart development. Regionalization of heart such as A, V and OFT is completed by 2 dpf. At 3 dpf, *elnb* is expressed specifically in OFT. Cardiac progenitor cells in OFT differentiate into smooth muscle cells and elastic and smooth muscle-composed BA is generated.

Taken together, our study permits us to propose the following scenario for the evolution and development of BA in the teleost; in the evolution of teleosts, the *elastin* gene was duplicated by the 3R WGD and one of the two paralogues, *elnb*, was relaxed from constraints of transcriptional regulation and changed its expression pattern to be restricted to BA (subfunctionalization). This relaxation was applied to not only transcriptional regulations but also to coding sequences in *elnb*, mutations were accumulated, and *elnb* acquired a new function (neofunctionalization, Fig. 5a). These modifications to the sequence of Elnb decreased elastic modulus and increased viscoelasticity, and may change cell fate determination of BA cells into smooth muscle cells (Fig. 5b). By these processes, the teleost acquired an evolutionary novel BA and sophisticated their single circulatory system, which played a central role in their adaptation to the aquatic environment and explosive radiation. This, again, illustrates a new mechanism for generating evolutionary novelty; alteration of the extracellular environment by a modified ECM alters cell fate determination due to mechanotransduction, which generates a new organ in evolution and development.

## Methods

### Collection of fish embryos and adult fish

Zebrafish (*D. rerio*) were maintained at 28.5 °C on a 14/10-h-light/dark cycle. Embryos were obtained from natural crosses of wild-type fish of the Tubingen AB background. Collected embryos were maintained at 28.5 °C, sorted into 1/3 Ringer media (39 mM NaCl, 0.97 mM KCl, 1.8 mM CaCl_2_, 1.7 mM HEPES at pH 7.2) and staged according to hpf at 28.5 °C and morphological criteria^57^. Medaka (*O. latipes*) were maintained at 28 °C on a 14/10-h-light/dark cycle. d-rR strains were used as wild type. Collected embryos were maintained at the same conditions as described above. Embryos were cultured and staged according to Iwamatsu^58^. Adult stickleback (*G. aculeatus*) were maintained in 342 l tanks with 10% seawater at 16 °C in the fish facility of the National Institute of Genetics (Mishima, Japan). Artificial insemination was conducted to obtain fertilized eggs, and the embryos were cultured in plastic plates (140 mm diameter) filled with 10% seawater at 16 °C. Staging was conducted according to a previous study^59^. *Polypterus* (*P. senegalus*) embryos were obtained from natural crosses of wild-type fish. Collected embryos were maintained at 28.5 °C, sorted into 1/3 Ringer media (39 mM NaCl, 0.97 mM KCl, 1.8 mM CaCl_2_, 1.7 mM HEPES at pH 7.2) and staged according to Takeuchi *et al.*^60^ Adult fish of *Polypterus* (*P. senegalus*), sturgeon (*A. gueldenstaedtii*), spotted gar (*L. oculatus*) and arowana (*O. bicirrhosum*) were purchased at aquarium shops in Tokyo, Japan. All embryos and adult fish were anaesthetized with 0.016% tricaine (MS-222, Sigma Chemical Co.) and fixed with 4% paraformaldehyde in PBS at the desired stages. All experimental procedures and animal care were performed according to the animal ethics committee of the University of Tokyo.

### Frozen sections

For cryosections, embryos and fish hearts were washed with PBS and embedded in 10% (w/v) sucrose/PBS solution at 4 °C overnight. Embryos were then placed in 30% (w/v) sucrose/PBS solution overnight at 4 °C. Embryos and fish hearts were placed in a 1:1 solution of 30% sucrose and optimal cutting temperature compound (Tissue-Tek, Torrance, CA) for 2 h at 4 °C, embedded in disposable moulds with optimal cutting temperature compound and frozen at −80 °C. Using a Leica CM 3050 S cryostat, we obtained 7 μm sections, which were mounted on glass slides and allowed to dry at room temperature.

### Elastica van gieson staining

For Elastica van Gieson staining, sectioned samples were washed in PBS and stained with Weigert's resorcin-fuchsin solution (Muto Pure Chemicals Co., Ltd) for 1 h. The sections were washed 3x with 100% ethanol for 3 min, then washed with running water for 5 min. After washing, samples were stained with Weigert's iron hematoxylin solution (Muto Pure Chemicals Co., Ltd) for 5 min then washed with running water for 10 min. Samples were then stained with van Gieson solution (Muto Pure Chemicals Co., LTD) for 5 min. After staining, samples were dehydrated and mounted.

### Immunohistochemistry

Immunohistochemistry was performed as described^41^ using the primary antibodies anti-α-sarcomeric actinin mouse monoclonal (1:200, Sigma, EA-53), anti-myosin light-chain kinase mouse monoclonal (1:200, Sigma, K36), muscle-specific MF20 (1:100, Developmental Studies Hybridoma Bank, University of Iowa, IA), and anti-phospho-Smad1/5/8 mouse polyclonal (1:200, Cell Signaling, #9511).

### Molecular phylogenetics

Peptide sequences with high similarities to coding sequences of zebrafish elastin and LIM domain kinase (limk) 1a/1b were retrieved from public databases using aLeaves^61^. The retrieved sequences were aligned with XCed, in which the multiple sequence alignment algorithm was implemented^62^. Using unambiguously aligned amino-acid sites, molecular phylogenetic trees were inferred preliminarily with the neighbour-joining method using XCed and finally with the maximum-likelihood method using PhyML version 3.0 (ref. 63). Among-site rate heterogeneity was taken into account by assuming a gamma distribution with the WAG model (WAG+G4+I). In both the neighbour-joining and maximum-likelihood methods, bootstrap resampling was performed with 100 replicates.

### Whole-mount *in situ* hybridization

Whole-mount *in situ* hybridization of zebrafish, medaka, stickleback and *Polypterus* embryos was performed as described previously^64^, except that the signals were visualized with BM Purple (Roche). Primer sequences for cloning genes of interest were as follows: zebrafish *elna* (primers: 5′-AGTTCTGCCTGGAGGTGGTC-3′, 5′-AAACAGTCCACCTGCACCTG-3′), zebrafish *elnb* (5′-CAGCAGGACTTGGAACAGGG-3′, 5′-ACCAAACCTGGGATCTCCTG-3′), medaka *elna* (primers: 5′-GCCAGGTGTGGGTGTTCCTG-3′, 5′-TCCTCCAGTTCCAAGTTGTC-3′), medaka *elnb* (primers: 5′-TGGACTTGGAAGCTACGGTG-3′, 5′-ATACCCTCCAGTTCCAGCAC-3′), stickleback *elna* (primers: 5′-CCTAAATATGGAGTCCCAGG-3′, 5′-ACTCCTGTTCCTAAACCTGC-3′), stickleback *elnb* (5′-CAGACCAGGTGGAGTGGTTC-3′, 5′-TGGTCCGAAGCCTCCTGTCC-3′), and *Polypterus eln* (primers: 5′-TCCTCCTGGCGCTGGAACTC-3′, 5′-GGTTTAAGTCCACCGAGTGC-3′).

### Morpholino and mRNA injections

Antisense MOs were designed to block translation and were purchased from Gene Tools, LLC (Philomath, OR, USA). The sequences of MOs used in this study were as follows: *elna* (5′-TGTGTAGCTGACATTGTTCTTTTGC-3′), *elnb* (5′-CCGGGCCATCCTGCTCTGTAATAAC-3′) and control (5′-CCTCTTACCTCAGTTACAATTTATA-3′, supplied as standard control morpholino oligo by Gene Tools, LLC.). Primer sequences for cloning genes of *Polypterus eln* used for rescue experiments are as follows: isoform1 (primers: 5′-ATGGCAAGCAGAAAGGCAGCGTCC-3′, 5′-ATTTTCTTTTTCTTCCACAGTATTGTCCTTGAGGAC-3′), isoform2 (primers: 5′-ATGGCAAGCAGAAAGGCAGCGTCC-3′, 5′-CTAGACCGTTAGTAGCCATATTATATGC-3′). mRNAs of *elna* and *elnb* were synthesized using the mMESSAGE mMACHINE *in vitro* transcription kit (Ambion) and purified using the RNeasy Mini kit (Qiagen). MOs and *in vitro* synthesized capped mRNA were dissolved in water to the desired concentration before injection and were injected into one-cell stage embryos. For the rescue co-injection experiment, each embryo was injected twice, first with the MO and then with the mRNA^65^.

### Generation of genetically mutated embryos by CRISPR/Cas9 system

The experiment was performed as describe previously^66^. Sequences of the genomic target sites and oligonucleotides for making the customized gRNA expression constructs are as follows: *elna* (primers: 5′-GATCAGTGTTGCGTTGCTCCTTCT-3′, 5′-TCGAAGAAGGAGCAACGCAACACT-3′), *elnb* (primers: 5′-GATCTCTTGCATGGATTTCTCCTGC-3′, 5′-TCGAGCAGGAGAAATCCATGCAAG-3′), *yap* (primers: 5′-GATCAACCAGCACAACCCTCCAGC-3′, 5′-TCGAGCTGGAGGGTTGTGCTGGTT-3′) and *tyrosinase* as a control (primers: 5′-GATCGGACTGGAGGACTTCTGGGG-3′, 5′-TCGACCCCAGAAGTCCTCCAGTCC-3′). Protospacer-adjacent motif search was conducted by Guide RNA Target Design Tool (Blue Heron, https://wwws.blueheronbio.com/external/tools/gRNASrc.jsp). Primers used for amplification of target region for T7 endnuclease I assay are as follows: *elna* (primers: 5′-CAACACCAGACATAGAAGAG-3′, 5′-CATGGTCACCTGCAGCACAC-3′), *elnb* (primers: 5′-TACGTAGTGTTCCTTCATGC-3′, 5′-CTATAACTGCAGTACACCTG-3′), and *yap* (primers: 5′-ATGCCTGCGTTAGAAGAGCC-3′, 5′-TGAAGAATGAGTCTGGCAGC-3′).

### Microscopy

Whole-mounted embryos were imaged on a Leica M205 FA automated fluorescence stereomicroscope with the imaging system AF6000 (Leica Microsystems, Wetzlar, Germany). Section samples were imaged on a BZ-9000 fluorescence microscope (Keyence, Osaka, Japan).

### Video microscopy

Movie images of beating zebrafish hearts were recorded as sequential phase-contrast images with a × 20 objective lens and × 0.7 conversion lens at a frame rate of −580 fps, a resolution of 1,024 × 512 pixels, and a depth of 8 bits using SI8000 imaging system (Sony Corporation, Tokyo, Japan).

### Motion vector analysis

Motion vectors of beating zebrafish hearts were obtained using a block-matching algorithm, as described previously^39^. Briefly, each frame was divided into square blocks of N × N pixels^39^,^40^. For a maximum motion displacement of *w* pixels per frame, the current block of pixels were matched to the corresponding block at the same coordinates in the previous frame within a square window of width N+2w. Here we set the parameters to *N*=16 and *w*=4, as determined empirically based on the throughput speed of calculation and accuracy of the block-matching detection. The best match on the basis of a matching criterion yielded the displacement of each block. The mean absolute error was used as the matching criterion. The matching function is given by


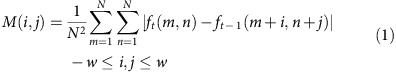


where *f*_*t*_(*m*, *n*) represents the intensity at coordinates (*m*, *n*) in the current block of *N* × *N* pixels, and *f*_*t*−1_(*m*+*i*, *n*+*j*) represents the intensity at new coordinates (*m*+*i*, *n*+*j*) in the corresponding block in the previous frame. We performed the above calculation for every 4 × 4 pixels in the frame with 1,024 × 512 pixels, and we obtained 32,768 motion vectors ((1,024 × 512 pixels)/(4 × 4 pixels)). The spatial average of the motion vector magnitude was defined by the following equation:


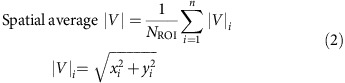


where |*V*|=absolute value of motion vector, *N*_ROI_=number of valid motion vectors in ROI, and *x*_*i*_ and *y*_*i*_ represent the components of the *i*th vector. By plotting the special average |*V*| against time, we can obtain information regarding the deformation speed of zebrafish hearts, that is, contraction and relaxation. Because we averaged the magnitude of the motion vectors as shown in the formula above, contraction and relaxation motion both gave positive values. In the present study, we simply termed ‘average speed' for the special average |*V*|.

### Analysis of the BA motion

We placed the ROI on the BA area of zebrafish hearts in each group, that is, control, *elna* MO and *elnb* MO. From the result of motion vector analysis, we distinguished the BA contraction and analysed the contraction duration and the averaged contraction deformation distance of the contraction (*N*=4).

### Dye labelling

DiI (2.5 mg ml^−1^ in dimethylsulphoxide) was injected into the anterior lateral plate mesoderm of zebrafish embryos at the 7-somite stage. The embryos were anaesthetized with 0.016% tricaine and embedded in 3% methylcellulose for stability during injection. After incubation, the embryos were again anaesthetized and placed in 3% methylcellulose during imaging at 4 dpf.

## Additional information

**Accession codes:** All nucleotide sequences reported in this study have been deposited in GenBank/EMBL/DDBJ under the accession codes LC099968 (*Oryzias latipes elna*), LC099969 (*Oryzias latipes elnb*), LC099970 (*Gasterosteus aculeatus elna*), LC099971 (*Gasterosteus aculeatus elnb*), LC099966 (*Polypterus senegalus eln* isoform1) and LC099967 (*Polypterus senegalus eln* isoform2).

**How to cite this article:** Moriyama, Y. *et al.* Evolution of the fish heart by sub/neofunctionalization of an *elastin* gene. *Nat. Commun.* 7:10397 doi: 10.1038/ncomms10397 (2016).

## Supplementary Material

Supplementary Information Supplementary Figures 1-5
