# Supplementary material for: Evolution of the fish heart by sub/neofunctionalization of an elastin gene
Source: Nat Commun. 2016 Jan 19;7:10397. doi: 10.1038/ncomms10397 (PMC4735684; doi:10.1038/ncomms10397)
Supplement: Supplementary Information — Supplementary Figures 1-5 [file ncomms10397-s1.pdf]

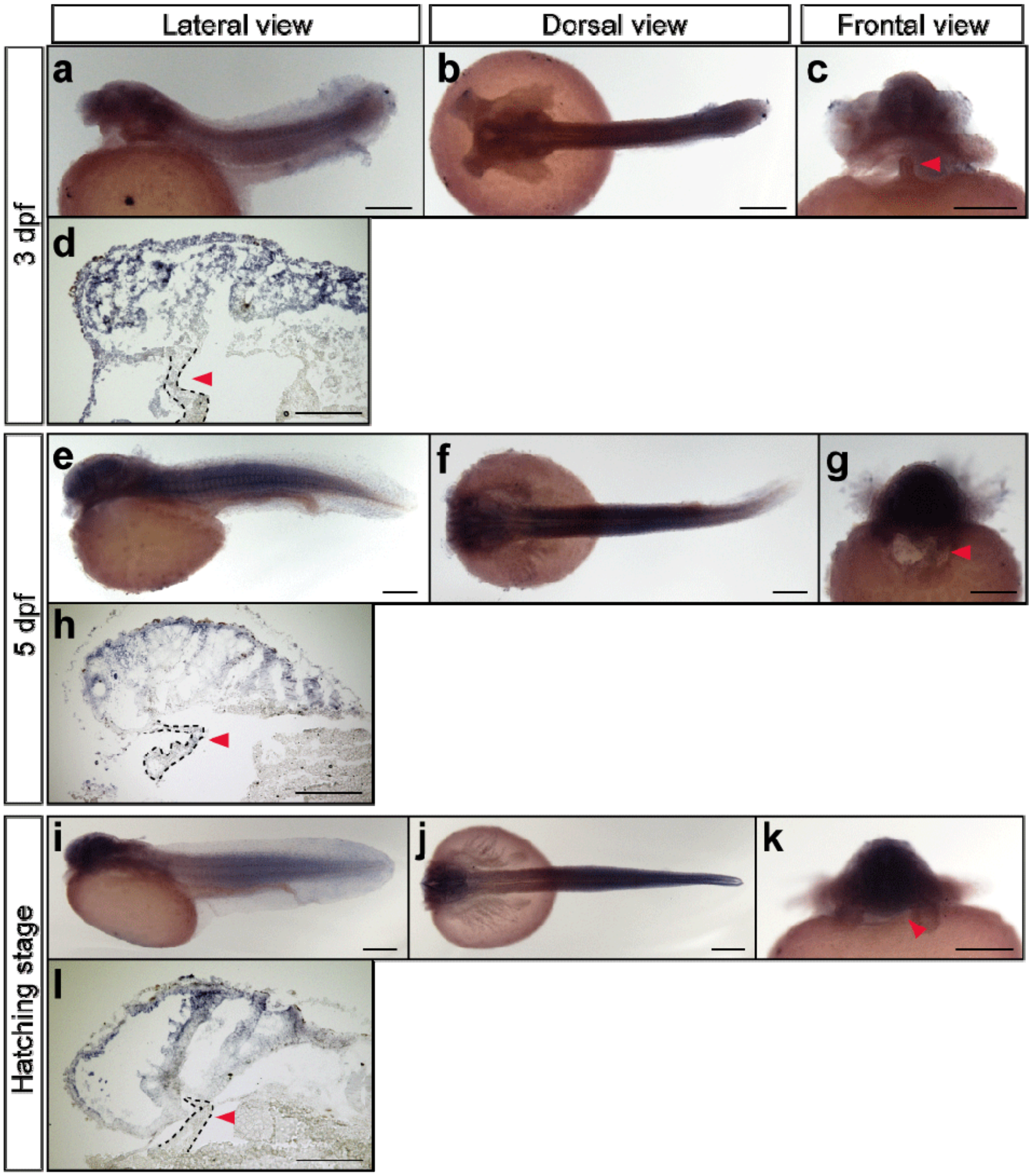

Supplementary Figure 1 | Expression pattern of *eln* in *Polypterus* embryos. Expression pattern of *eln* in 3 (**a-d**), 5 (**e-h**) dpf and hatching stage (**i-l**) embryos from lateral (**a, e, i**), dorsal (**b, f, j**) and frontal (**c, g, k**) view. Scale bars, 500 μm. (**d, h, l**) Sagittal section of *in situ* hybridization embryos. Arrowheads indicate hearts. Dashed line depicts outline of heart tubes. Scale bars, 300 μm.

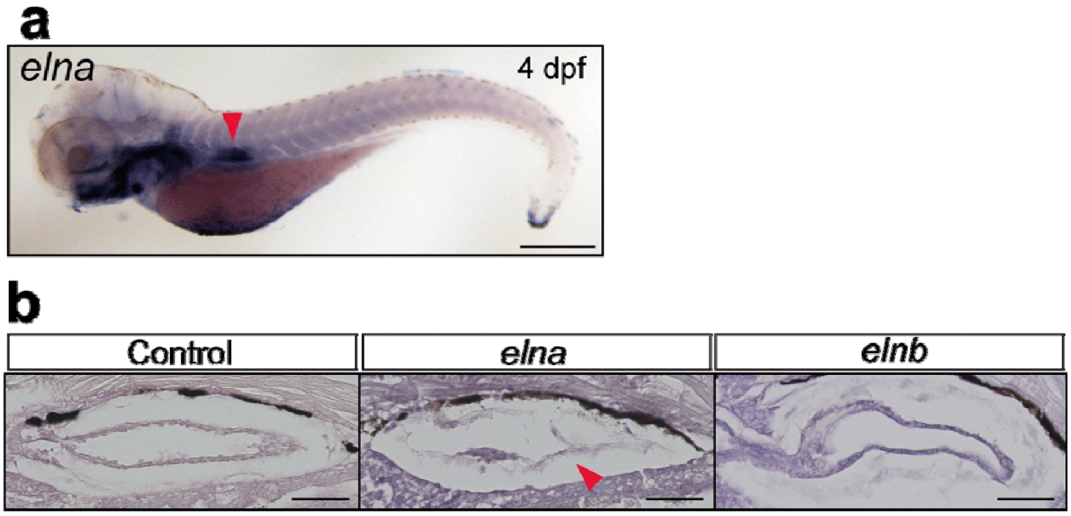

Supplementary Figure 2 | Expression pattern of *elna* in zebrafish 4 dpf embryos. (**a**) Expression pattern of *elna* in 4 dpf embryos. Arrowhead indicates *elna* expression in swimbladder. Scale bar, 400 μm. (**b**) Swimbladder morphologies in control, *elna* morphant and *elnb* morphant embryos. Arrowhead indicates swimbladder defect in *elna* morphants. Scale bars, 50 μm.

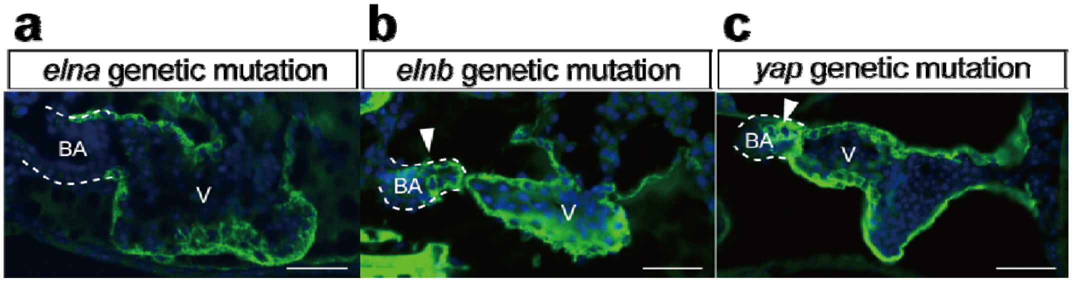

Supplementary Figure 3 | BA morphology in *elna*, *elnb* and *yap* genetic mutants. BA morphology in *elna* (**a**), *elnb* (**b**) and *yap* (**c**) genetic mutants. Arrowheads indicate ectopic cardiomyocytes in BA. BA, bulbus arteriosus; V, ventricle. Scale bars, 50 μm.

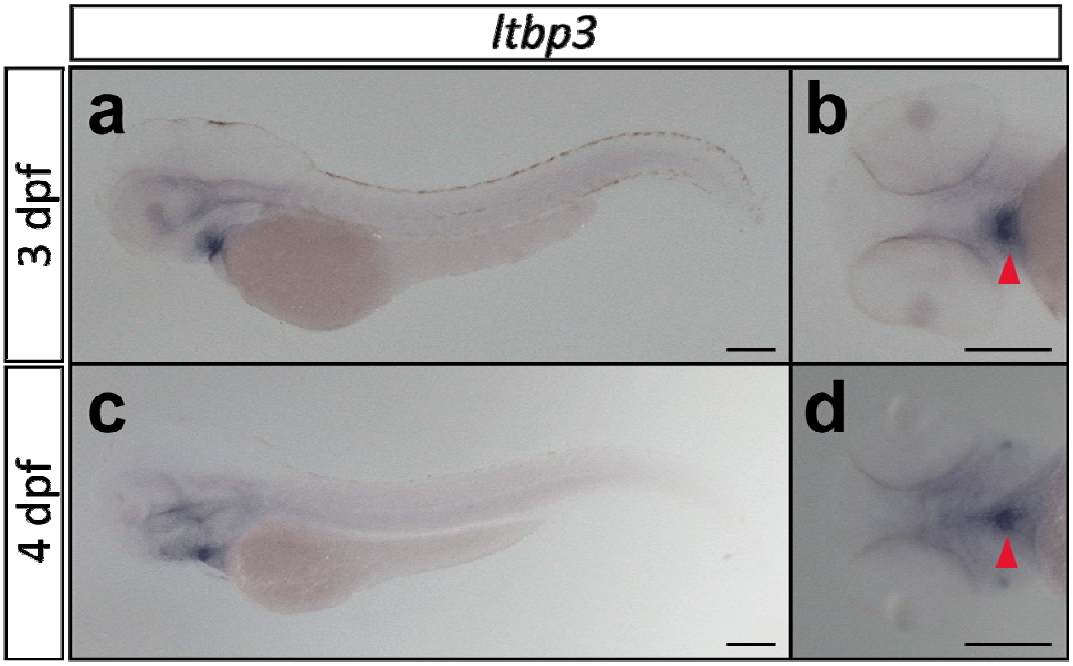

Supplementary Figure 4 | Expression pattern of *ltbp3* in zebrafish embryos. Expression patterns of *ltbp3* in 3 (**a, b**) and 4 (**c, d**) dpf embryos. Arrowheads indicate *ltbp3* expression in BA. Scale bars, 200 µm.

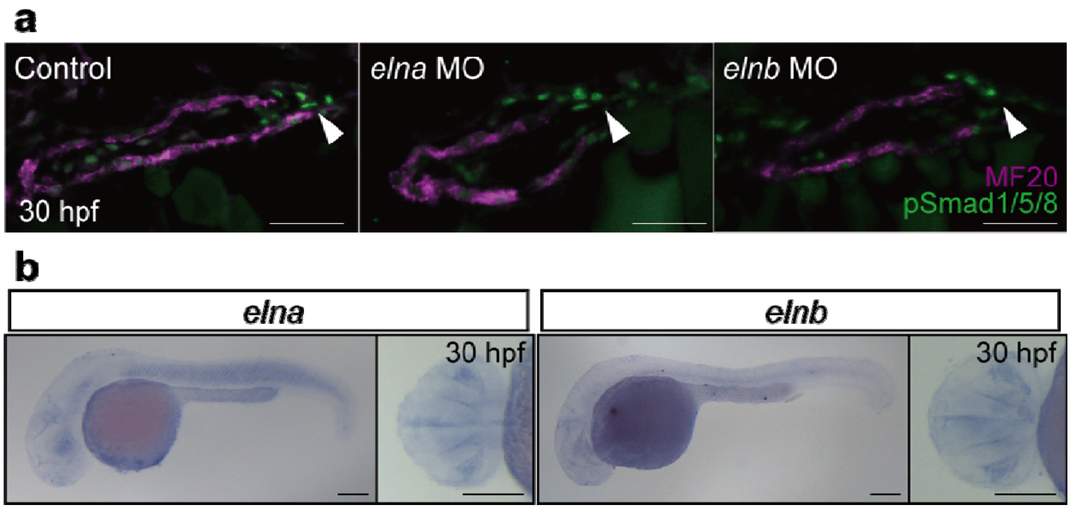

Supplementary Figure 5 | Phosphorylation level of Smad1/5/8 and expression pattern of *elna* and *elnb* in 30 hpf zebrafish embryos. (**a**) Phosphorylation levels of Smad1/5/8 at 30 hpf in control, *elna* morphants and *elnb* morphants. pSmad1/5/8 at arterial pole (arrowheads) are not changed in *elna* and *elnb* morphants. Scale bars, 50 µm. (**b**) Expression pattern of *elna* and *elnb* in 30 hpf embryos. *elna* and *elnb* are not expressed in the heart at 30 hpf. Scale bars, 200 µm.
